# NK Cells Negatively Regulate CD8 T Cells to Promote Immune Exhaustion and Chronic *Toxoplasma gondii* Infection

**DOI:** 10.3389/fcimb.2020.00313

**Published:** 2020-07-08

**Authors:** Daria L. Ivanova, Ryan Krempels, Stephen L. Denton, Kevin D. Fettel, Giandor M. Saltz, David Rach, Rida Fatima, Tiffany Mundhenke, Joshua Materi, Ildiko R. Dunay, Jason P. Gigley

**Affiliations:** ^1^Molecular Biology, University of Wyoming, Laramie, WY, United States; ^2^Department of Immunology and Microbiology, University of Colorado Anschutz Medical Campus, Aurora, CO, United States; ^3^Institute of Inflammation and Neurodegeneration, Otto-von-Guericke Universität Magdeburg, Magdeburg, Germany

**Keywords:** *Toxoplasma gondii*, NK cells, CD8 T cell exhaustion, chronic infection, ILC

## Abstract

NK cells regulate CD4+ and CD8+ T cells in acute viral infection, vaccination, and the tumor microenvironment. NK cells also become exhausted in chronic activation settings. The mechanisms causing these ILC responses and their impact on adaptive immunity are unclear. CD8+ T cell exhaustion develops during chronic *Toxoplasma gondii* (*T. gondii*) infection resulting in parasite reactivation and death. How chronic *T. gondii* infection impacts the NK cell compartment is not known. We demonstrate that NK cells do not exhibit hallmarks of exhaustion. Their numbers are stable and they do not express high PD1 or LAG3. NK cell depletion with anti-NK1.1 is therapeutic and rescues chronic *T. gondii* infected mice from CD8+ T cell exhaustion dependent death, increases survival after lethal secondary challenge and alters cyst burdens in brain. Anti-NK1.1 treatment increased polyfunctional CD8+ T cell responses in spleen and brain and reduced CD8+ T cell apoptosis in spleen. Chronic *T. gondii* infection promotes the development of a modified NK cell compartment, which does not exhibit normal NK cell characteristics. NK cells are Ly49 and TRAIL negative and are enriched for expression of CD94/NKG2A and KLRG1. These NK cells are found in both spleen and brain. They do not produce IFNγ, are IL-10 negative, do not increase PDL1 expression, but do increase CD107a on their surface. Based on the NK cell receptor phenotype we observed NKp46 and CD94-NKG2A cognate ligands were measured. Activating NKp46 (NCR1-ligand) ligand increased and NKG2A ligand Qa-1b expression was reduced on CD8+ T cells. Blockade of NKp46 rescued the chronically infected mice from death and reduced the number of NKG2A+ cells. Immunization with a single dose non-persistent 100% protective *T. gondii* vaccination did not induce this cell population in the spleen, suggesting persistent infection is essential for their development. We hypothesize chronic *T. gondii* infection induces an NKp46 dependent modified NK cell population that reduces functional CD8+ T cells to promote persistent parasite infection in the brain. NK cell targeted therapies could enhance immunity in people with chronic infections, chronic inflammation and cancer.

## Introduction

*Toxoplasma gondii* (*T. gondii*) is an obligate intracellular protozoan that is the 3rd leading cause of foodborne illness in the U.S. (Mead et al., 1999) At least one-third of the human population is infected with this parasite and it is a major health concern for people who become immune compromised and in the developing fetus (Harms Pritchard et al., 2015; Gigley, 2016). Presently, there are no vaccines or drugs available to prevent or eliminate this infection and infection with this parasite is life long (Coppens, 2014; Radke et al., 2018). *T. gondii* infection induces a potent cell mediated response that is initiated by the production of IL-12 which helps activate CD8+ T cells to produce IFNγ (Suzuki and Remington, 1988; Suzuki et al., 1988; Gazzinelli et al., 1994a,b). CD8+ T cell IFNγ production is the major mediator of this infection. Despite induction of a robust Th1 response, the parasite is never cleared. The immunological reason why this infection is not cleared is still unknown.

In mouse models of chronic *T. gondii* infection the parasite can spontaneously reactivate causing the development of toxoplasmic encephalitis (TE) and death (Bhadra et al., 2011b). Parasite reactivation has been attributed to the development of immune exhaustion of parasite specific CD8+ T cells (Bhadra et al., 2011a,b, 2012; Hwang et al., 2016). The CD8+ T cells in mice harboring chronic *T. gondii* infection exhibit immune exhaustion characteristics similar to persistent viral infections (Wherry and Kurachi, 2015). Loss of activated CD8+ T cells resulting in a reduced functional cell population, expression of high levels of programmed death 1(PD1) and increased apoptosis of CD8+ T cells. This loss of functional CD8+ T cells results in parasite reactivation and death of the animals. Importantly, the exhausted CD8+ T cells can be rescued with anti-PDL1 therapy during chronic *T. gondii* infection and this also prevents parasite reactivation and death. The mechanisms underlying the development of CD8+ T cell exhaustion and dysfunction during chronic *T. gondii* infection are still unclear.

NK cells are innate lymphoid cells (ILCs) that provide early cytotoxicity and cytokine dependent protection during infections and cancer (Geiger and Sun, 2016). NK cells are important for control of acute *T. gondii* infection (Denkers et al., 1993; Johnson et al., 1993) and are activated early during parasite infection by IL-12 (Gazzinelli et al., 1993; Hunter et al., 1994). As a result of IL-12 signaling, NK cells produce high levels of IFNγ, which helps control the parasite prior to T cell activation. NK cells are more complex than previously thought and appear to not only be activated and work as a component of innate immunity during acute infections, but may also continue to work along side CD4+ and CD8+ T cells during the adaptive phase of immunity. NK cells have been shown to acquire memory-like features after exposure to haptens, during viral infections and after cytokine stimulation (O'Leary et al., 2006; Cooper et al., 2009; Sun et al., 2009; Paust et al., 2010). This highlights their ability to not simply fall into the background once adaptive immunity is established, but also to continue to play a role in immunity after acute infections are resolved. NK cells have also been shown to become exhausted (Gill et al., 2012; Sun et al., 2015; Alvarez et al., 2019; Zhang et al., 2019). This can occur in the tumor microenvironment, chronic stimulation and persistent HCV infection. In these different disease situations, NK cells become dysfunctional and as a result could contribute to the persistence of infections and reduced clearance of tumor cells. NK cells can also be negative regulators of the adaptive response during acute infections and cancer. Through several interactions including TRAIL, NKp46 and yet to be defined receptors, NK cells can lyse CD4+ and CD8+ T cells resulting in less effective adaptive responses thereby promoting pathogen and tumor persistence (Lang et al., 2012; Waggoner et al., 2012; Cook and Whitmire, 2013; Peppa et al., 2013; Crouse et al., 2014; Schuster et al., 2014). In addition, NK cells produce IL-10 during acute systemic infections including *T. gondii* infection dampening the activation of adaptive immune responses (Perona-Wright et al., 2009). Much of what is known about the development of these other non-protective NK cell responses is in the acute disease or infection setting and less in known about how NK cells behave during chronic infections long after acute infection is resolved.

Based upon the knowledge that CD8+ T cells become exhausted to promote *T. gondii* persistence, NK cells can remain active for long periods of time, NK cells have the potential to become exhausted and they can regulate development of adaptive immune responses we were interested to test how chronic *T. gondii* infection impacted the NK cells and how did NK cells impact the outcomes of chronic toxoplasmosis. Our results indicate that NK cells are still present during chronic *T. gondii* infection. They do not exhibit characteristics of immune exhaustion. They contribute to the loss of exhausted CD8+ T cells and their removal helps maintain control of chronic *T. gondii* infection. We also demonstrate that NK cells develop a unique phenotype that supports the hypothesis that NKp46 recognition of ligand and loss of NKG2A interaction with Qa-1b promotes the development of an NK cell population that negatively regulates CD8+ T cell function contributing to parasite reactivation and death. Our data highlight that NK cells could be therapeutic targets to enhance long-term immunity to chronic *T. gondii* infection.

## Materials and Methods

### Mice

C57BL/6 (B6), B6.129S6-IL-10^tm1Flv^/J (IL-10-GFP Tiger) mice were purchased from The Jackson Laboratory. All animals were housed under specific pathogen-free conditions at the University of Wyoming Animal Facility. This study was carried out in strict accordance following the recommendations in the Guide for the Care and Use of Laboratory Animals of the National Institutes of Health. The University of Wyoming Institutional Animal Care and Use Committee (IACUC) (PHS/NIH/OLAW assurance number: A3216-01) approved all animal protocols.

### *T. gondii* Parasites and Infection

Tachyzoites of RH were cultured by serial passage in human fetal lung fibroblast (MRC5, ATCC) cell monolayers in complete DMEM (supplemented with 0.2 mM uracil for CPS strain). For mouse infections, parasites were purified by filtration through a 3.0-μm filter (Merck Millipore Ltd.) and washed with phosphate-buffered saline (PBS). Mice were infected intraperitoneally (i.p.) with 1 × 10^3^ or 1 × 10^6^ RH tachyzoites or 1 × 10^6^ CPS tachyzoites. The brains of CBA mice 5 weeks after ME49 infection were used as a source of ME49 cysts. Mice were infected i.p. or i.g. (intragastrically) with 10 or 200 ME49 cysts.

### NK Cell Depletion and NKp46 Blockade *in vivo*

To deplete NK cells, B6 mice were treated i.p. with 200 μg of anti-NK1.1 (PK136, Bio X Cell). To block NKp46 mice were treated i.p. with 50 μg non-depleting LEAF purified anti-NKp46 (29A1.4, Biolegend) (Narni-Mancinelli et al., 2012). Antibody treatments were started 5 weeks after infection with ME49 and continued every other day for 2 weeks for flow cytometry assays or until non-treated animal groups died from reactivation of *T. gondii*. No treatment animals were treated with 200 μl of 1 X PBS i.p.

### Brain and Spleen T Cell Isolation and Stimulation

Single-cell suspensions of brain and spleen were prepared from mice. To harvest brain lymphocytes and assess their phenotype and function, mice were anesthetized and perfused with 20 ml of 0.9% saline with heparin as described (Donley et al., 2016). Brains were then homogenized in 1 X PBS using a dounce homogenizer. Brains were pelleted by centrifugation then homogenates were added to 30% percoll^®^ and centrifuged at 2,000×g for 20 min at 15°C to collect lymphocytes from the pellet. Brain lymphocytes were then plated at 0.5–1.5 × 10^6^ cells/well in complete Iscove's DMEM medium (10% FBS, Na Pyruvate, non-essential amino acids, penicillin, β-2 Mercaptoethanol) (Corning). 0.5 × 10^6^ congenically marked CD45.1 splenocytes were also added to the brain lymphocyte wells as feeder cells for antigen restimulation. Spleens were crushed through 70 μm cell strainers (VWR) in 1 X PBS. Splenocytes were then treated with 3 ml of RBC lysis buffer for 3 min at 37°C to lyse erythrocytes, washed then resuspended in complete Iscoves DMEM. Spleen cells were plated at 1 × 10^6^ cells per well. Brain and spleen cells were then pulsed with 20 μg/ml Toxoplasma lysate antigen (TLA) for 8 h and cultured at 37°C in 5% CO_2_. After 8 h, 1X protein transport inhibitor cocktail (PTIC) containing Brefeldin A/Monensin (eBioscience, Thermo Fisher Scientific) with or without anti-CD107a (eBio1D4B, eBioscience, Thermo Fisher Scientific) was added to each well in complete Iscove's DMEM medium (Corning). After 4 h incubation at 37°C in 5% CO_2_, cells were prepared for flow cytometry.

### ILC Functional Assays

For ILC function assays, spleen cells were stimulated for 4 h with plate bound anti-NK1.1 in the presence of 1 × protein transport inhibitor cocktail (PTIC) containing Brefeldin A/Monensin (eBioscience, Thermo Fisher Scientific) and anti-CD107a (eBio1D4B, eBioscience, Thermo Fisher Scientific) in complete Iscove's DMEM medium (Corning). Cells were incubated during stimulation at 37°C in 5% CO_2_ for 4 h. Cells were then first surface stained then intracellularly stained to measure function. ILC phenotypes were measured directly *ex vivo*. Spleen cells were stained following procedures indicated below after fixable Live/Dead staining (Invitrogen).

### Flow Cytometry

Single cell suspensions from brain or spleen were assayed for immune cell phenotype and functions. Phenotype assays were performed directly *ex vivo* after harvest. Function assays were performed after antigen pulse cells or stimulation. All flow cytometry staining was performed using the same procedure for all experiments. Cells were washed twice with PBS and stained for viability in PBS using Fixable Live/Dead Aqua (Invitrogen) for 30 min. After the cells were washed with PBS, surface staining was performed using antibodies diluted in stain wash buffer (2% fetal bovine serum in PBS and 2 mM EDTA) for 25 min on ice in the presence of 2.4G2 FcR blockade to reduce non-specific staining. For phenotype analysis cells were then fixed for 10 min using fixation/permeabilization solution (BD biosciences). For functional assays after fixable live/dead and surface staining, the cells were fixed and permeabilized for 1 h on ice in Fixation/Permeabilization solution (BD Bioscience), followed by intracellular staining in 1 X permeabilization wash buffer (BD Bioscience) with anti-IFNγ and anti-granzyme B (XMG1.2, NGBZ, eBioscience, Thermo Fisher Scientific) for 45 min. Antibodies used for surface staining were against: CD3 (17A2), CD49b (DX5), CD49a (HMα1), NKp46 (29A1.4), NK1.1(PK136), CD4 (RM4-5), CD8b (YTS156.7.7), KLRG1 (2F1/KLRG1), 2B4 (m2B4), Ly49I (YLI-90), Ly49H (3D10), CD94 (18d3), NKG2AB6 (16A11), LAG3 (C9B7W), PD1 (29F.1A12), PDL1(10F.9G2), CD107a (1D4B), CD45.1 (A20), CD45.2 (104). These antibodies were from Biolegend. Anti-Qa-1b (6A8.6F10.1A6) was from eBiosciences and anti-Ly49D (4E5) was from BD Biosciences. NKp46 (NCR1) ligand was stained using the soluble NKp46 receptor fused to human Fc (NCR1-hFc, RND systems). Bound soluble receptor was then detected using a secondary antibody anti-human IgG. To assess apoptosis, cells were also stained using Annexin V (Annexin V staining kit, Biolegend). The cells were resuspended in 1 X PBS and analyzed using Guava easyCyte 12HT flow cytometer (Millipore-SIGMA) and FlowJo software (Tree Star).

### Parasite and Cyst Burdens

Cyst burdens were quantified using microscopy. Brains of mice infected with Type II strain ME49 were harvested and homogenized with a dounce homogenizer in 2 ml of 1 X PBS. Ten microliter of homogenized brain was placed onto a microscope slide and covered with a cover slip. Microscope slides were examined and cysts in the homogenate were counted. A minimum of 5 slides per mouse was counted.

### Survival Studies

WT B6 mice were infected with 10 cysts i.g. of the type II parasite strain ME49. After 5 weeks of infection mice were treated or not i.p. with 200 μg anti-NK1.1 (PK136, BioXCell) or 50 μg LEAF purified anti-NKp46 (Biolegend). Mouse treatments were performed every other day until completion of experiments. Mice were monitored daily for morbidity and mortality. Mice were evaluated on a 1–5 scale with 5 indicating highest morbidity. Mice reaching a level 5 score are not moving, severely hunched, eyes shut and not eating or drinking. Mice were sacrificed prior to death and after they reached level 5 clinical score for no more than 24 h. For survival experiments after rechallenge, ME49 infected animals were treated or not with anit-NK1.1 (PK136, BioXcell). After the 2nd dose of anti-NK1.1 mice were challenged with either a lethal dose 200 cysts of ME49 i.g. or 1,000 tachyzoites of the type I highly virulent strain RH i.p. ILC depletion was continued every other day as in other experiments. Control mice were uninfected naïve B6 mice only given the challenge infection (either ME49 or RH). Survival of chronically infected rechallenged mice were monitored and assessed on the 1–5 scale as described above.

### Statistical Analysis

Statistical analysis was performed using Prism 7.0d (GraphPad) and Microsoft Excel 2011. Significant differences were calculated using either unpaired Student's *t*-test with Welch's correction, Mann-Whitney nonparametric test or analysis of variance (ANOVA). The log-rank (Mantel-Cox) test was used to evaluate survival rate. Data is presented in graphs as the mean± standard deviation (SD). Significance is denoted as follows: ns, not significant (*p* > 0.05) or significant with a maximum *p*-value of 0.05 or less.

## Results

### NK Cell Exhaustion

**Figure 1:**
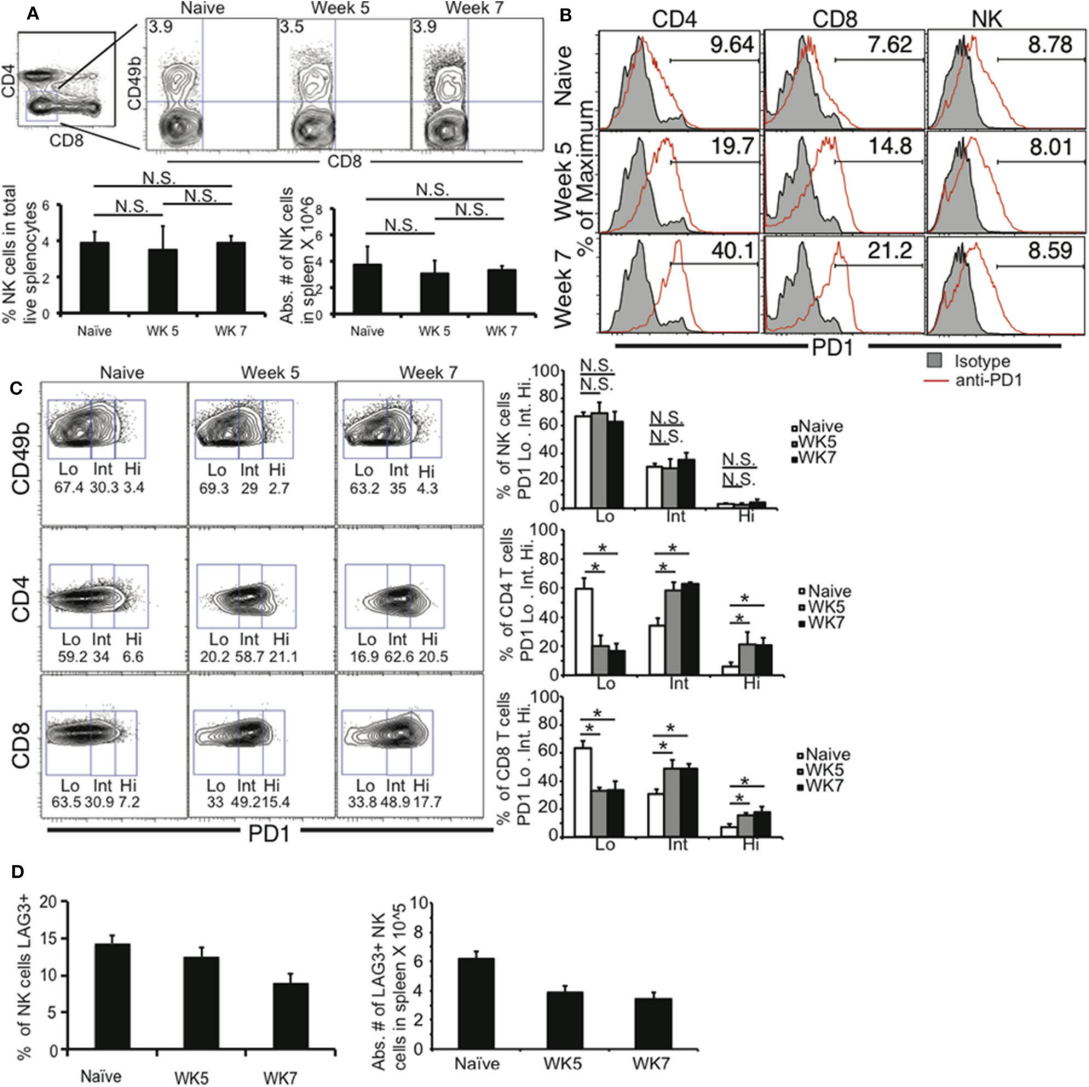
NK cells do not exhibit immune exhaustion characteristics during chronic *T. gondii* infection. C57BL/6 mice were orally infected or not with 10 cysts of ME49 and 5 and 7 weeks after infection spleen cells were analyzed for **(A)** NK cell (Lineage-CD49b+) frequency and number. NK cells, CD4+ and CD8+ T cells were then analyzed for **(B)** PD1 MFI and **(C)** PD1+ frequency based on separating the populations into PD1 low (Lo), intermediate (Int), and high (Hi). NK cells were then assayed for **(D)** LAG3 expression. Raw flow data presented are representative and based on means from 3 independent experiments. All graphs are mean ± SD. Graphs present pooled data from 2 independent experiments. Significance is denoted by ^*^with a *p* ≤ 0.05, *n* = 4–5 mice per group.

Previous studies have demonstrated that during chronic *T. gondii* infection, CD4+ and CD8+ T cells develop immune exhaustion resulting in their dysfunction (Bhadra et al., 2011b, 2012; Hwang et al., 2016). This ultimately results in the death of B6 mice beginning in the late chronic stage of infection (~WK 7 post infection) due to parasite reactivation. Parasite reactivation is detected by loss of cyst numbers in the brains and increased parasitemia in the blood. To further dissect the immune mechanisms contributing to T cell exhaustion during chronic *T. gondii* infection, we investigated the role of innate lymphoid cells and more specifically NK cells. NK cells can participate in immune responses long after the innate response has transitioned into the adaptive response (O'Leary et al., 2006; Sun et al., 2009). NK cells acquire characteristics of memory. NK cells can also develop characteristics of immune exhaustion in the tumor microenvironment (Gill et al., 2012). They traffic to tumor sites, have reduced numbers, effector function and upregulate PD1 expression on their surface. Based on the ability of NK cells to contribute to immunity after the innate response is over and their potential to develop immune exhaustion we determined whether NK cells were still in abundance during late chronic *T. gondii* infection and their immune exhaustion status. Mice were infected with 10 cysts of the Type II *T. gondii* strain ME49, known to induce T cell exhaustion during long-term infection. At week 5 and 7 post infection spleens were harvested and NK cell frequencies and numbers were measured using flow cytometry. Lineage negative (CD4–CD8–) cells were analyzed for CD49b+ cells. As shown in Figure 1A, the frequencies and absolute numbers of splenic NK cells (CD4–CD8–CD49b+) did not significantly decrease from week 5 to 7 post infection. As previously published week 7 is when CD4+ and CD8+ T cells decrease in both frequency and number (Bhadra et al., 2011b; Hwang et al., 2016). An increase in Programmed death 1 (PD1) on T cells is a hallmark of immune exhaustion. During late chronic *T. gondii* infection, both CD4+ and CD8+ T cells have been reported to increase their PD1 expression leading to loss of function of these T cells and parasite reactivation (Bhadra et al., 2011b; Hwang et al., 2016). To further assess whether NK cells exhibited characteristics of immune exhaustion during late chronic *T. gondii* infection we measured their PD1 expression. As shown in Figure 1B, the mean fluorescence intensity (MFI) of PD1 increased on both CD4+ and CD8+ T cells, however, NK cells did not increase their expression of PD1. In addition, the frequencies of CD4+ or CD8+ T cell PD1 high (PD1 Hi), PD1 intermediate (PD1 Int) both increased significantly at week 5 and 7 post infection (Figure 1C). The frequencies of PD1 Hi or Int did not change on NK cells over the course of infection. Another marker of exhaustion is lymphocyte activating gene 3 (LAG3) expression. LAG3 increases on CD4+ and CD8+ T cells during late chronic *T. gondii* infection (Hwang et al., 2016). We did not detect an increase in LAG3 expression (Figure 1D) on NK cells during chronic *T. gondii* infection. Based on the results splenic NK cells do not appear to decrease in number or express PD1 or LAG3 at high levels compared to CD4+ and CD8+ T cells during chronic *T. gondii* infection.

### NK Cell Role in Chronic *T. gondii* Infection

**Figure 2:**
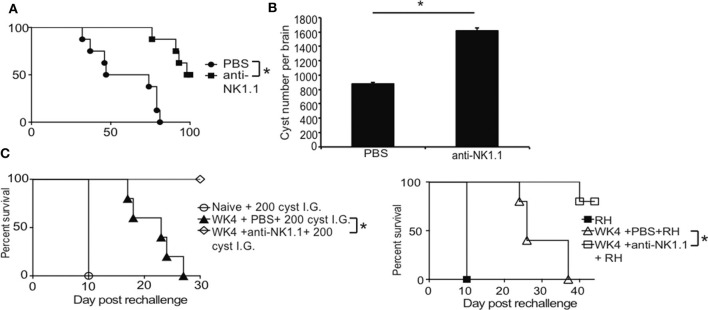
NK cells promote immune exhaustion and parasite reactivation during chronic *T. gondii* infection. C57BL/6 mice were infected orally with 10 cysts of ME49 and infection outcome monitored. **(A)** Survival after 200 μg anti-NK1.1 or 200 μl 1 X PBS every 2nd day starting at week 5 p.i. Graph presents pooled data from 3 independent experiments, *n* = 4–5 mice per group **(B)** Brain cyst burden after anti-NK1.1 or 1 X PBS treatment starting at week 5 p.i. Graph presents data from 1 experiment repeated independently 2 times with *n* = 5 mice per group. **(C)** Survival of chronically infected animals after lethal secondary 200 ME49 cyst or 1,000 tachyzoite RH strain challenge with or without anti-NK1.1 treatment. Experiments were repeated independently 2 times with *n* = 4–5 mice per group. All graphs are mean ± SD. ^*^denotes significance as *p* ≤ 0.05.

Based on results of our studies, NK cells did not appear to develop characteristics of immune exhaustion raising the question about how might NK cells contribute to immune control of *T. gondii* during chronic infection. WT B6 mice typically succumb to spontaneous reactivation of the parasite in the CNS and die (Bhadra et al., 2011b). Parasite reactivation in the brain can be observed via reemergence of parasitemia and a gradual decline of cyst numbers in the brain. Interestingly, treatment with anti-PDL1 antibody rescues animals from death and slows down parasite reactivation resulting in higher cyst numbers in the CNS in a CD8+ T cell dependent manner (Bhadra et al., 2011a,b, 2012). To begin to address how NK cells are behaving during chronic *T. gondii* infection NK cells were depleted using anti-NK1.1 in mice starting at week 5 post infection. Mice were treated every other day until the experiment was terminated at 100 days post infection. Mice with NK cells began to succumb to the infection around week 7 (49 days) post infection (Figure 2A). Mice treated with anti-NK1.1 did not start to succumb to the infection until 80 days post infection. All mice with NK cells were dead by 80 days post infection whereas 50% of mice depleted of their NK cells were still alive at 100 days post infection (Figure 2A). We next measured whether cyst burdens in the brain were maintained better when NK cells were depleted because as previously published, cyst numbers decrease in the brain as the parasite reactivates due to immune exhaustion and rescuing exhausted CD8+ T cells with anti-PDL1 maintains higher cyst numbers in the brains (Bhadra et al., 2011a,b, 2012, 2013). As shown in Figure 2B, mouse brain cyst burdens were higher in mice that were depleted of NK cells than mice with NK cells. To test how NK cells were impacting secondary immune control of the parasite, B6 mice were infected with ME49 and at 5 weeks depleted of their NK cells or not with anti-NK1.1. 2 days after start of treatment, mice were challenged with a lethal dose of either 200 cysts of ME49 i.g. or 1,000 tachyzoites of RH i.p. and monitored for survival. As shown in Figure 2C, mice with their NK cells succumbed to challenge significantly earlier than mice with NK cells depleted. These results suggest that NK cells appear to have a negative affect on long-term immunity to chronic *T. gondii* infection modifying cyst levels in the brain and reducing the effectiveness of adaptive recall responses.

### NK Cell Impact on CD8+ T Cell Responses

**Figure 3:**
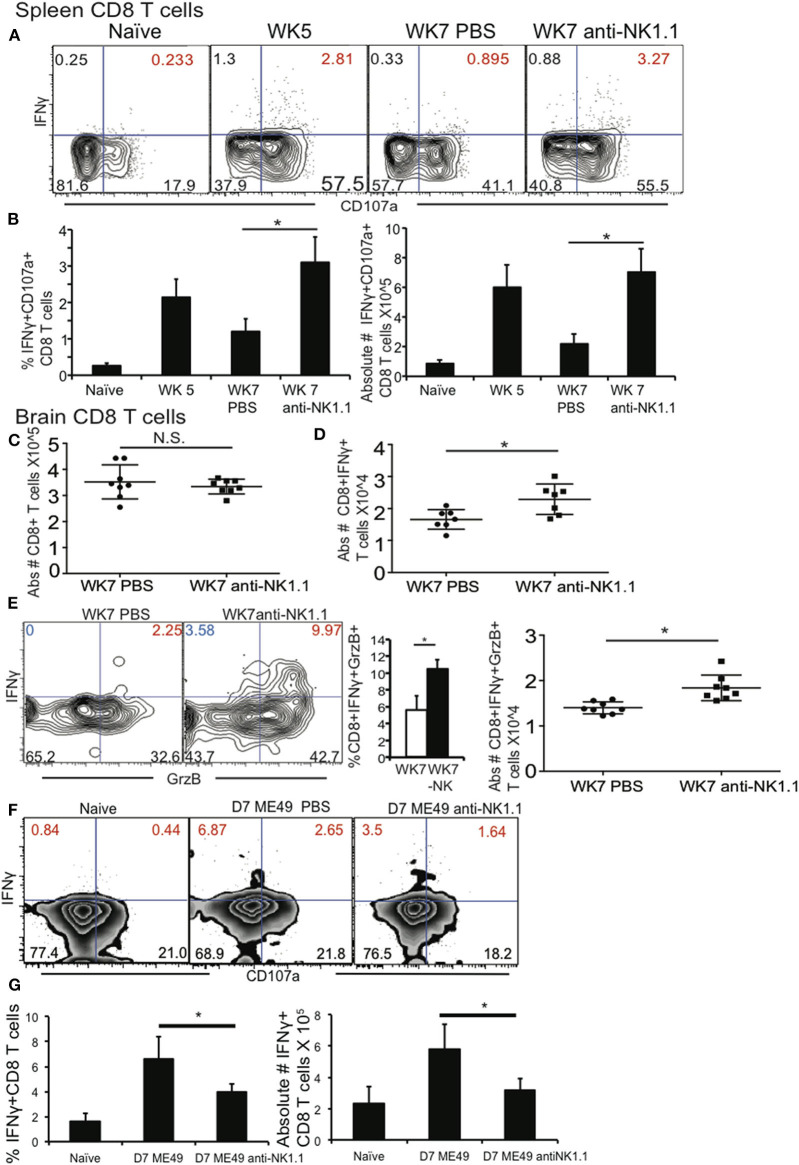
NK cells reduce polyfunctional CD8+ T cells by increasing their apoptosis during chronic *T. gondii* infection. C57BL/6 mice were infected as described and 5 weeks after infection treated with 200 μg anti-NK1.1 or 200 μl 1 X PBS i.p. Brain and spleen cells were harvested and restimulated *ex vivo* with TLA. **(A)** Contour plots present spleen CD8+ T cells analyzed for IFNγ+ × CD107a+. **(B)** Graphs present frequency and absolute # of polyfunctional IFNγ+CD107a+CD8+T cells. Data presented is from 1 experiment repeated independently 2 times with *n* = 5 mice per group. **(C)** Graphs present pooled data from 2 independent experiments of absolute numbers of brain CD8+ T cells with or without anti-NK1.1 treatment starting at wk 5 post infection. **(D)** Graphs present pooled data from 2 independent experiments of absolute numbers of brain IFNγ+ CD8+ T cells from animals treated with 200 μg anti-NK1.1 or 1 X PBS i.p. **(E)** Contour plots of brain IFNγ+GrzB+CD8+ T cells (red numbers are frequency) are presented. Graphs present the frequency and absolute number of brain polyfunctional (IFNγ+Grzb+) CD8+ T cells from 2 independent experiments with *n* = 4 mice per group. **(F,G)** B6 mice were treated or not with 200 μg of anti-NK1.1 starting at D-1 then infected with 10 cysts of ME49 strain i.g. Mice were treated every other day with anit-NK1.1. On day 7 post infection animals were sacrificed and spleen cells isolated then stimulated with TLA and stained for polyfunctionality (IFNγ+CD107a+). **(F)** Contour plots present CD8+ T cells stained for IFNγ+ × CD107a+ during acute *T. gondii* infection. **(G)** Graphs present pooled data from 2 experiments showing frequency (%) and absolute number of IFNγ+ CD8+ T cells in spleen. *N* = 4 mice per group. All graphs are mean ± SD. ^*^denotes significance with a *p* ≤ 0.05.

**Figure 4:**
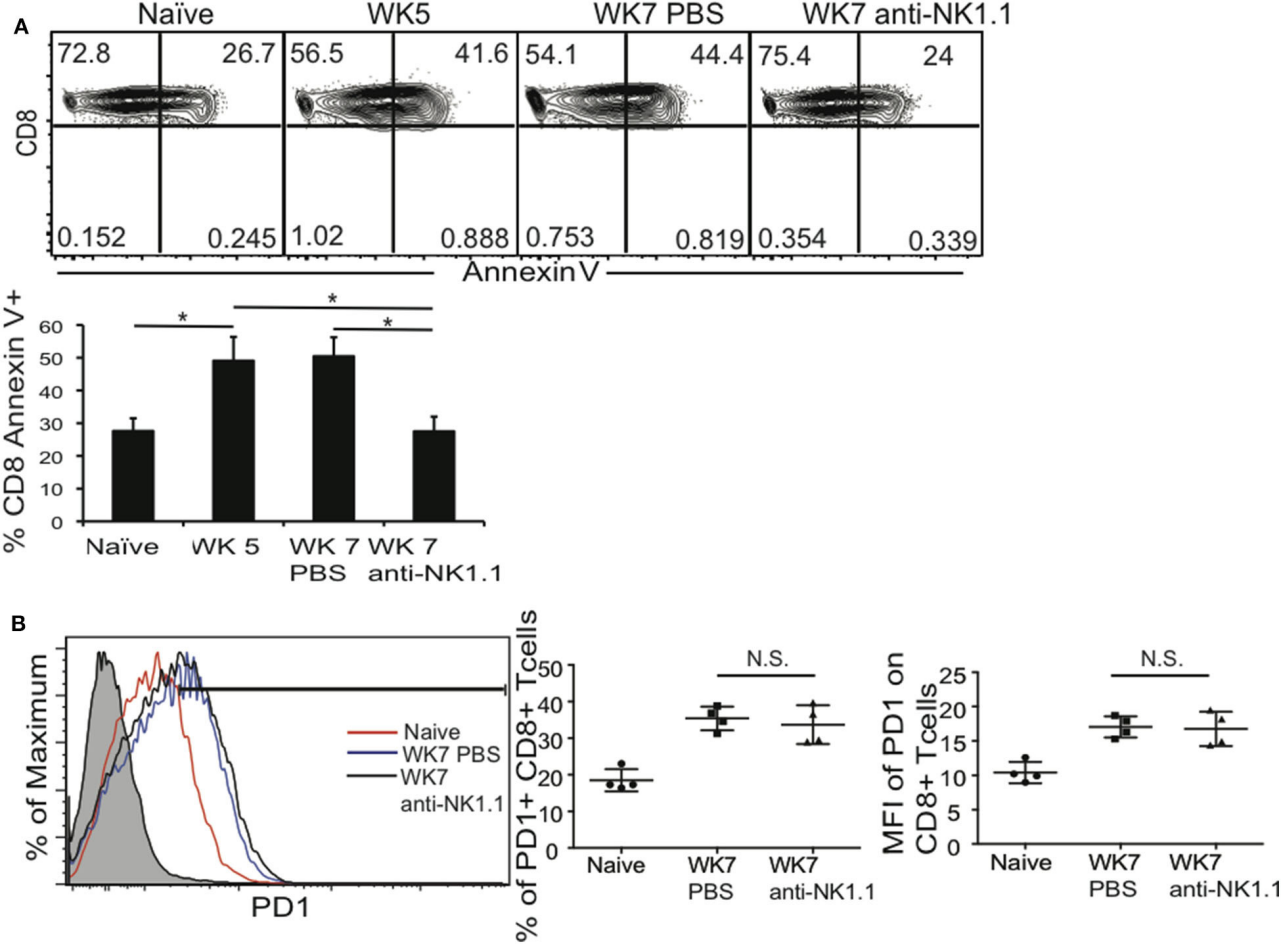
NK cells increase CD8+ T cells apoptosis during chronic *T. gondii* infection. C57BL/6 mice were infected and some groups at week 5 after infection were treated with anti-NK1.1 or 1 X PBS i.p. as described. Week 5, week 7 non-treated and week 7 treated infected mice were sacrificed and spleen cells isolated for CD8+ T cell Annexin V staining and PD1 and compared to naïve animals. **(A)** Contour plots present frequency of Annexin V+ CD8+ T cells and graphs present mean ± SD. **(B)** Histogram presents PD1 expression on splenic CD8+ T cells from anti-NK1.1 treated or not treated mice. Graphs present frequency and MFI of PD1 on CD8+ T cells. Data presented are from 1 experiment repeated 2 independent times with *n* = 4 mice per group. Significance is denoted by ^*^with a *p* ≤ 0.05 non-significant results are denoted with N.S.

Parasite reactivation and mouse death during chronic *T. gondii* infection occurs when parasite specific CD8+ T cells develop immune exhaustion (Bhadra et al., 2011b). Their exhaustion results in decreased frequency and number of polyfunctional (IFNγ+CD107a+, or IFNγ Granzyme B+) CD8+ T cells in the periphery and CNS. Since depletion of NK cells during chronic *T. gondii* resulted better survival we measured how this impacted polyfunctional CD8+ T cells in the spleen and brain. Infected mice were treated starting at week 5 post infection with anti-NK1.1 or 1 X PBS for 2 weeks as in previous experiments and spleen and brain cells were assayed for the frequency and absolute number of polyfunctional CD8+ T cells. As shown in Figure 3A, NK cell depletion of week 5 infected mice resulted in the maintenance of the frequency and absolute number of IFNγ+CD107a+ CD8+ T cells. In the brain, NK cell depletion starting at week 5 post infection did not significantly impact overall CD8+ T cell numbers, however, NK cell depletion resulted in a significant increase in the absolute numbers of IFNγ+ CD8+ T cells and in the frequency and absolute numbers of IFNγ+GrzB+ CD8+ T cells (Figures 3C–E). Several studies have demonstrated that during acute viral infections (MCMV, LCMV) infection, NK cells are negative regulators of priming of adaptive immune responses (Lang et al., 2012; Cook and Whitmire, 2013; Crouse et al., 2014; Schuster et al., 2014; Waggoner et al., 2014; Cook et al., 2015; Rydyznski et al., 2015). This negative regulation promotes viral persistence and immune exhaustion of the T cells. However, during acute *T. gondii* infection previous studies suggest that NK cells could be positive regulators of the priming of adaptive immune responses against the parasite (Combe et al., 2005; Goldszmid et al., 2007, 2012; Guan et al., 2007). Many of the earlier *T. gondii* studies used anti-asialo GM1 antibody to deplete NK cells without knowing that this antibody targets not only NK cells, but effector populations of CD8+ T cells (Ivanova, 2019). We tested whether NK cells provided a different function during acute *T. gondii* infection and promoted priming of CD8+ T cells as compared to NK cells in chronic *T. gondii* infection, which appear to inhibit CD8+ T cell function. B6 mice were treated with anti-NK1.1 1 or 1 X PBS alone a day prior to infection with ME49 strain of *T. gondii* and then infected with 10 cysts i.g. NK depleted mice were treated with anti-NK1.1 for 6 days and on day 7 all mice were harvested and their spleen CD8+ T cell functionality was measured. As shown in Figures 3F,G, CD8+ T cells were activated by day 7 post infection, however, NK cell depleted animals had significantly fewer activated CD8+ T cells (IFNγ+CD107a+ plus IFNγ+CD107a–) than mice that still have their NK cells. Thus, during acute infection NK cells are important for priming CD8+ T cells to protect against infection, but during chronic *T. gondii* infection, NK cells change their function and negatively regulate CD8+ T cells to promote parasite reactivation and mouse death. To further define why polyfunctional CD8+ T cells were reduced during chronic *T. gondii* infection in the presence of NK cells, we measured CD8+ T cell apoptosis using Annexin V staining and assessed CD8+ T cell PD1 expression in the presence or absence of NK cells. As presented in Figure 4A, CD8+ T cell apoptosis was significantly increased in chronically infected mice at week 5 and 7 post infection in the spleen. NK cell depletion significantly reduced the level of CD8+ T cell apoptosis in the spleen of chronically infected mice at week 7 post infection. NK cells also did not appear to impact the frequency of PD1+ CD8+ T cells or level of PD1 expression on CD8+ T cells in chronically infected mice (Figure 4B). NK cells appear to contribute to CD8+ T cell exhaustion during chronic *T. gondii* infection possibly by increasing CD8+ T cell apoptosis, but not by increasing their PD1 expression.

### NK Cell Phenotype During Chronic *T. gondii* Infection

**Figure 5:**
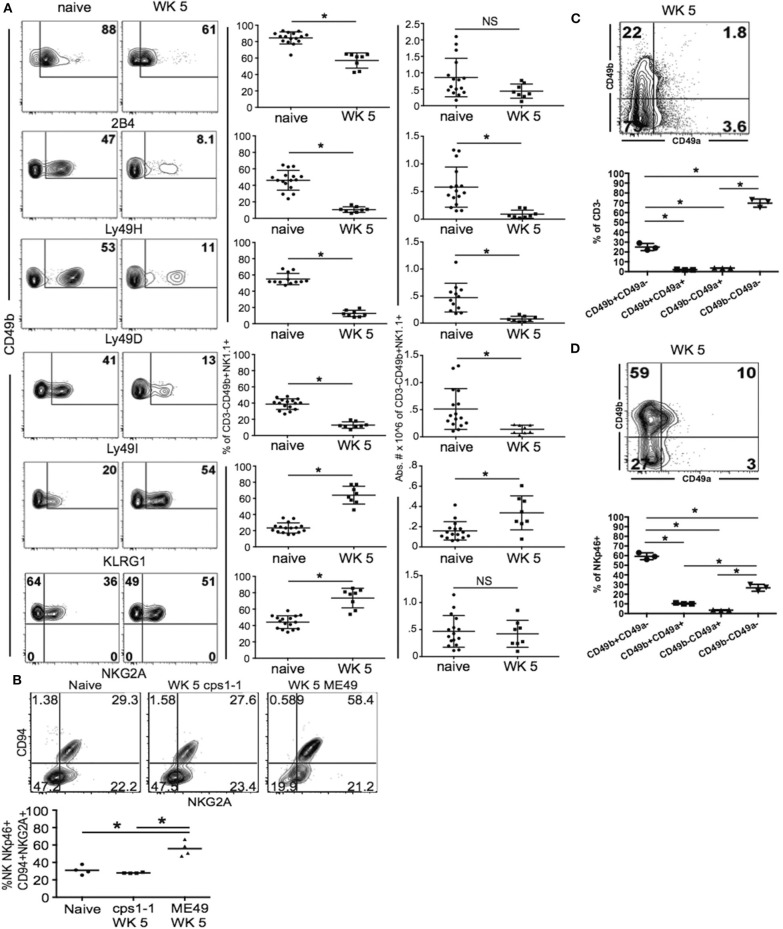
NKp46+NKG2A+KLRG1+ NK cells are enriched in spleen during chronic *T. gondii* infection. C57BL/6 mice were orally infected with 10 cysts of ME49 and analyzed for the NK cell receptors 2B4, Ly49H, Ly49D, Ly49I, KLRG1, CD94, and NKG2A by flow cytometry. **(A)** Contour plots present the frequency of CD49b+ × receptor + cells comparing naïve animals to week 5 post infection. Graphs show pooled data from 3 experiments of frequency and absolute number of CD49b+ Receptor+ cells. **(B)** Mice were infected with either 1 × 10^6^ tachyzoites of *csp1-1* i.p. or 10 cysts ME49 i.g. At week 5 post infection, lineage-CD49b+NKp46+ cells were analyzed for CD94 × NKG2A. Contour plots present data from one experiment showing frequency of CD94 × NKG2A cell populations. The graph presents data from 1 experiment comparing the frequency of CD94+NKG2A+ cells between naïve, *cps1-1* and ME49 mice. **(C)** Mice were infected with 10 cysts of ME49 i.p. then spleen cells were analyzed at week 5 post infection for CD49a × CD49b to identify ILC1 compared to NK cells. Contour plot presents the frequency of CD49a × CD49b cells in the CD3– population. **(D)** Contour plot presents the frequency of CD49a × CD49b cells in the CD3–NKp46+ population. **(C,D)** Graphs present the frequency of CD49b+CD49a–, CD49b+CD49a+, CD49b–CD49a+, and CD49b–CD49a–. All experiments were repeated independently a minimum of 2 times with *n* = 3–4 per group. ^*^denotes significance with a *p* ≤ 0.05.

Published studies indicate that NK cells use several mechanisms to regulate adaptive immune responses (Perona-Wright et al., 2009; Lang et al., 2012; Peppa et al., 2013; Crouse et al., 2014; Schuster et al., 2014; Xu et al., 2014; Crome et al., 2017; Kwong et al., 2017). Many of these mechanisms rely on the expression of specific NK cell receptors, which allow the NK cells to target specific adaptive immune cell populations and induce their apoptosis, lysis or suppression. NK cell receptors are also very important for normal protective NK cell functions including Ly49H, which recognize m157 of MCMV (Lanier, 2005; Sun et al., 2009). These specific interactions promote the enrichment of NK cell subpopulations expressing specific receptor combinations as was demonstrated for memory-like NK cells during MCMV infection. During acute *T. gondii* infection, we have previously published that there does not appear to be a dominant NK cell population activated. *T. gondii* infection may only induce cytokine dependent NK cell activation resulting in global activation of a large array of different IFNγ producing and protective NK cell subpopulations (Ivanova et al., 2016). In the studies presented here, NK cells appear to change their function to promote CD8+ T cell compartment dysfunction and alter levels of chronic infection. This suggests the NK cell compartment could be modified and a specific NK cell subpopulation develops to erode immunity to the parasite during chronic *T. gondii* infection. To begin to define what NK cell receptors might be involved in contributing to CD8+ T cell exhaustion during chronic *T. gondii* infection, we performed an exhaustive assessment of NK cell receptor expression during week 5 chronic *T. gondii* infection. As shown in Figure 5A, the NK cell compartment had significantly reduced frequencies of cells that expressed 2B4, Ly49H, Ly49D, and Ly49I. This was observed on lineage—CD49b+ cells in the spleen. We did not detect any differences in TRAIL expression (data not shown). We observed significant increases in the frequencies of NK cells (lin-CD49b+) that expressed KLRG1 and NKG2A (Figure 5A). The number of NK cells expressing KLRG1 also increased significantly during chronic *T. gondii* infection. The significant increase in KLRG1+ NK cells and NKG2A+ NK cells suggested these cells were being enriched within the NK cell compartment. A recent study suggests that CD49a+ ILC1 may develop from NK cells and exhibit this phenotype in the liver of mice infected with the vaccine strain of *T. gondii cps1-1* and a different limited cyst forming type II strain Prugniaud (Park et al., 2019). Therefore, to determine if this was also occurring in NK cells in the spleens after infection with the vaccine strain, we infected mice with *cps1-1* and 5 weeks later assayed the spleen cells for CD94+NKG2A+ NK cells. As shown in Figure 5B, *cps1-1* strain parasites did not induce the same increase in frequency of CD94+ NKG2A+ NK cells as did ME49. To further investigate whether ILC1 were enriched in the NK cell compartment, we measured the frequencies of ILC1 (CD49a+CD49b–) compared to NK cells (CD49a–CD49b+) in the CD3– and CD3– NKp46+ cell populations in the spleens of chronically infected mice. As shown in Figure 5C, NK cells (CD49b+CD49a–) comprised 22% of the CD3– population of cells and 60% of the lineage negative NKp46+ population of cells. ILC1 were present at a very low level. Therefore, in the spleen, NK cells appear to be the dominant population of ILC present and they are enriched for a specific receptor phenotype, which is Lin—CD49b+ CD49a–NKp46+ CD94+ NKG2A+KLRG1+.

### NK Cell Function During Chronic *T. gondii* Infection

**Figure 6:**
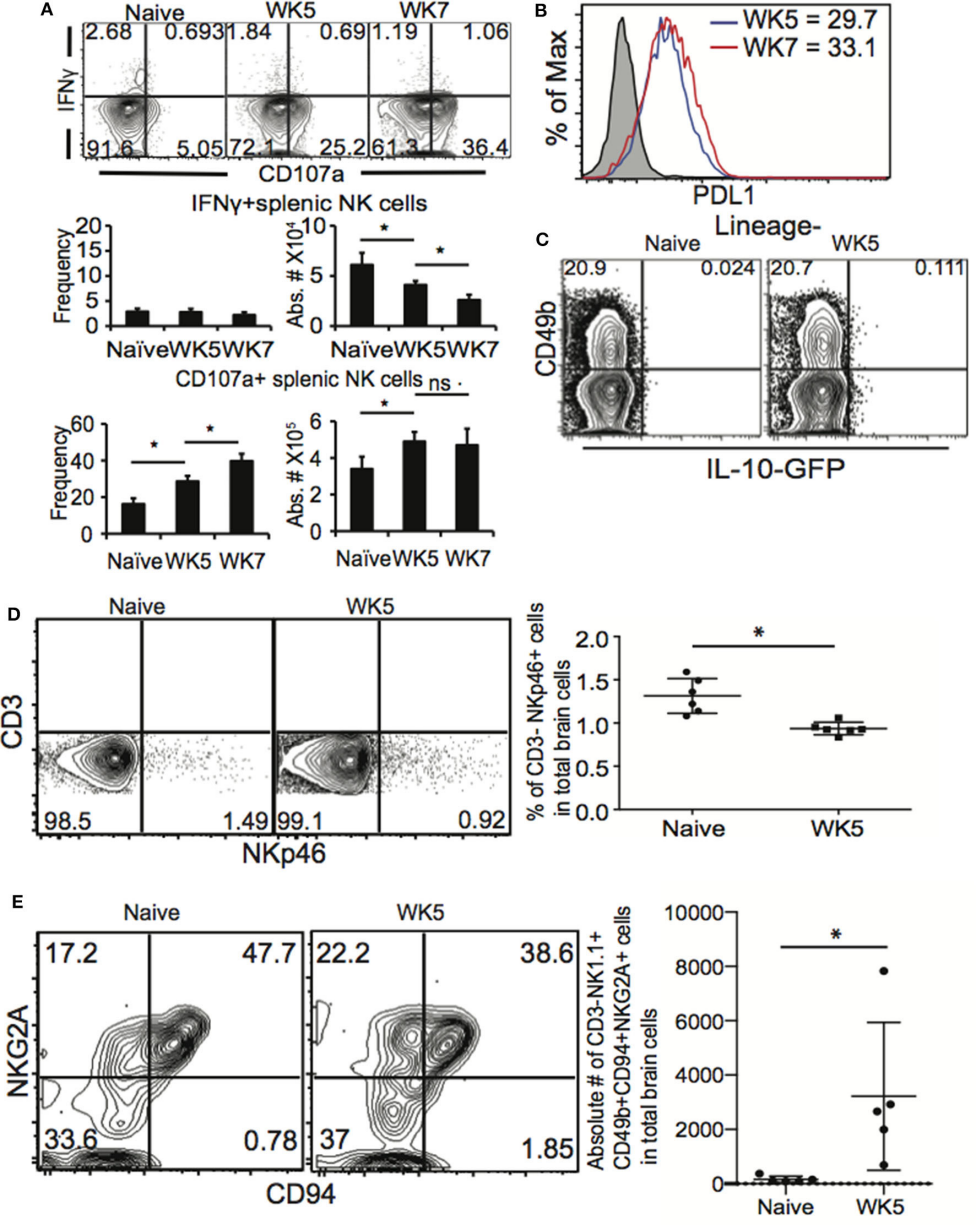
NK cells have altered function during chronic *T. gondii* infection. C57BL/6 or IL-10 reporter TIGER-GFP mice were orally infected with 10 cysts of ME49 and 5 and/or 7 weeks after infection spleen cells analyzed for function. **(A)** Spleen cells were stimulated *ex vivo* with plate bound anti-NK1.1 then stained for NK cells (CD3–CD49b+ NKp46+) IFNγ and CD107a. Contour plots present frequency data gated on NK cells and compares IFNγ × CD107a. Graphs present the frequency and absolute number of IFNγ+ NK cells (top graphs) and CD107a+ NK cells (bottom graphs). Graphs present mean ± SD. **(B)** Splenic NK cells were assayed for PDL1 expression. Histogram presents the MFI of PD-L1 on NK cells from weeks 5 and 7 post infection mice. **(C)** Contour plots present the frequency of IL-10 GFP+ NK cells in naïve compared to week 5 post infection mice. **(D)** Brain cells were isolated and stained for lineage markers, CD49b and NKp46. Contour plots present the frequency of CD3–NKp46+ cells in the CNS. Graphs present the pooled data from 2 experiments of frequency of CD3–NKp46+ cells in the CNS with *n* = 3 mice per group. **(E)** Brain cells form naïve and WK5 infected mice were stained for CD3–CD49b+NK1.1+CD94+NKG2A+ cells. Contour plots present representative data from 1 experiment repeated 2 times with *n* = 5 mice per group showing CD3–CD49b+NK1.1+ cells analyzed for the frequency of CD94 and NKG2A+ cells. The graph presents absolute numbers of CD94+NKG2A+ cells in total NK cells from brains. Significance is denoted by ^*^with a *p* ≤ 0.05.

NK cells are the cytotoxic cells of the ILC lineage (Diefenbach et al., 2014; Eberl et al., 2015). NK cells are also capable of producing high levels of IFNγ upon activation. ILC1 are not cytotoxic and produce high levels of IFNγ upon activation. During acute *T. gondii* infection NK cells and ILC1 are known to produce IFNγ in an IL-12 dependent manner (Denkers et al., 1993; Gazzinelli et al., 1993; Hunter et al., 1994, 1995; Klose et al., 2014) We have recently demonstrated that after vaccination, NK cells respond a second time to help control challenge infection by producing IFNγ in an IL-12 and IL-23 dependent manner (Ivanova et al., 2019). We observe that NK cells during chronic *T. gondii* infection modify their role in immunity to the parasite and are not protective, but detrimental. They also express an altered receptor repertoire that suggests enrichment for a specific cell phenotype. Therefore, to begin to investigate how NK cells are negative regulators of CD8+ T cells during chronic *T. gondii* infection we first assayed their function. Mice were infected as above and starting at week 5 post infection we assessed NK cell (CD3–CD49b+ NKp46+) function (IFNγ × CD107a) by flow cytometry. As shown in Figure 6A, after *ex vivo* stimulation, naïve NK cells were capable of producing both IFNγ and expressing the surrogate cytotoxicity marker CD107a. However, after week 5 of infection (Figure 6A), NK cells produced very little IFNγ while significantly increasing their CD107a expression. This pattern of function was observed also at week 7 post infection. Increases in CD107a+ NK cells were observed in both frequency and absolute number. Interestingly, the frequency of IFNγ+ NK cells continued to decrease from weeks 5 to 7 post infection. The data shown in Figure 6A was generated *ex vivo* by using plate bound anti-NK1.1 crosslinking. We repeated *ex vivo* analysis using PMA/Ionomycin and still the NK cells did not produce IFNγ (data not shown). Interestingly, if these cells were ILC1, we would have expected them to produce IFNγ. We next measured whether the NK cells expressed PD-L1, the ligand for PD1. As shown in Figure 6B, splenic NK cells did not appear to increase their expression of PD-L1 as PD-L1 MFI was not significantly different between weeks 5 and 7 post infection. During acute systemic *T. gondii* infections, NK cells have been shown to produce IL-10 (Perona-Wright et al., 2009). Therefore, we obtained IL-10GFP TIGER reporter mice and infected them with 10 cysts of ME49 i.g. Comparing naïve to week 5 post infected mice (Figure 6C), we did not observe any IL-10 production by NK cells. A recent study demonstrated that NKp46+ ILC could contribute to the development of neurodegenerative disease by being in the CNS and promoting Th17 responses (Kwong et al., 2017). We next determined how chronic *T. gondii* infection impacted NK cells in the CNS. Mice were infected as previously described and at week 5 post infection, mice were perfused, brains dissected and immune cells isolated. Cells were analyzed for CD3–NKp46+ (Figure 6D) and CD3–CD49b+NK1.1+CD94+NKG2A+ (Figure 6E) populations. As shown in Figure 6D, we did not observe an increase in frequency of CD3– Nkp46+ NK cells in the CNS of *T. gondii* chronically infected mice at week 5 post infection. There appeared to be a decrease. However compared to naïve animals, we observed an increase in absolute number of CD3–CD49b+NK1.1+CD94+NKG2A+ NK cells in the brains of chronically infected mice, a similar phenotype to splenic NK cells. Our investigation of the function of the NK cells acting as negative regulators of immunity during chronic *T. gondii* infection suggests that NK cells have reduced IFNγ production, but may increase cytotoxicity. They do not produce IL-10 and similar to splenic NK cell phenotypes are enriched for CD94+ NKG2A+ population in the CNS of chronically infected mice.

### NKp46 and NKG2A NK Cells During Chronic *T. gondii* Infection

**Figure 7:**
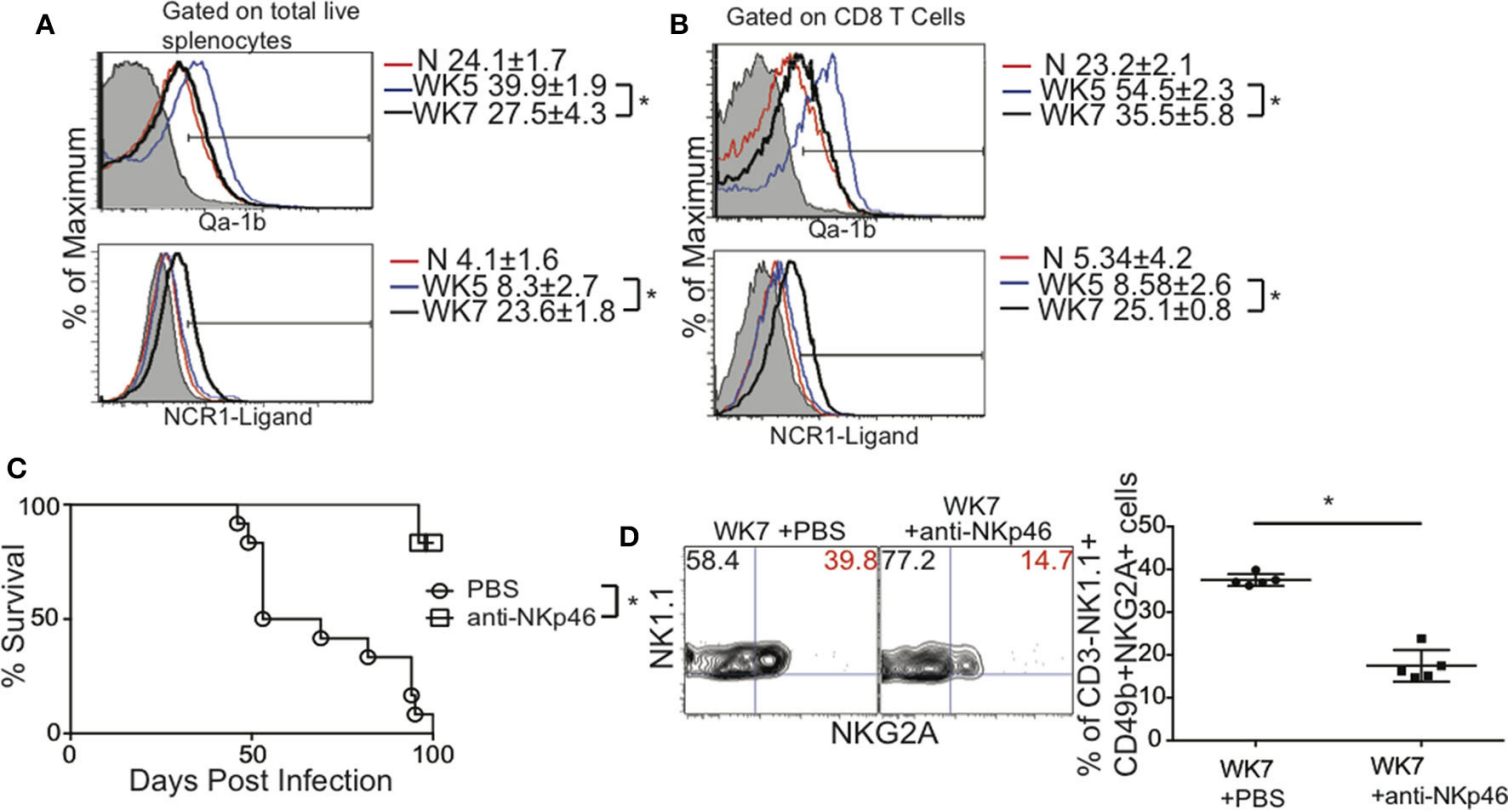
Blockade of NKp46 rescues mice from death caused by CD8+ T cell exhaustion induced parasite reactivation. C57BL/6 mice were orally infected as above and total splenocytes were assayed for NKG2A ligand Qa-1b and NKp46 ligand using soluble NCR1 fused to human Ig Fc. **(A)** Histograms present the MFI ± SD of QA-1b (top) and NCR1-ligand (bottom) from total splenocytes. **(B)** Histograms present the MFI ± SD of QA-1b (top) and NCR1-ligand (bottom) from CD8+ T cells. **(C)** Mice were infected with 10 cysts of ME49 i.g. and treated with 50 μg of anti-NKp46 or 1 X PBS i.p. starting at week 5 p.i. Mice were treated every other day for the duration of this experiment. The survival graph presents pooled data from 3 independent experiments with *n* = 4–5 mice per group. The log-rank (Mantel-Cox) test was used to evaluate survival rates. **(D)** Mice were infected with 10 cysts of ME49 i.g. and treated with 50 μg of anti-NKp46 or 1 X PBS i.p. starting at week 5 p.i. Mice were treated every other day for the duration of this experiment and harvested 2 weeks after start of treatment. The frequency of CD3–NK1.1+CD49b+NKG2A+ NK cells was measured in the spleen. Contour plots and graph present frequency of NK1.1+NKG2A+ spleen cells from 1 experiment repeated independently twice with *n* = 5 mice per group. ^*^denotes significance with *p* ≤ 0.05.

The NK cell phenotype we observed Lin—CD49b+ CD49a–NKp46+ CD94+ NKG2A+KLRG1+ suggest that NKp46 and NKG2A may contribute NK cell negative regulation of the immune response to *T. gondii* in chronically infected mice. This is based on the concept of NK cell licensing (Kim et al., 2005). NK cell licensing determines the responsiveness of NK cells to self vs. non-self. A licensed NK cell expresses both activating and inhibitory receptors on its surface and as a result is tuned or permitted to respond when self is absent. An absence or reduction in self, usually reduced MHC expression can be detected on a target cell. At the same time, increases in non-self detected by elevated ligands binding to the activating receptor activate the NK cell. NKp46 is an activating receptor expressed on NK cells, ILC1 and some ILC3 (Eberl et al., 2015; Cortez and Colonna, 2016). The ligand NKp46 recognizes is not very well-described. Potential ligands for NKp46 vary in source and structure and to date may include Influenza virus HA, Sigma 1 protein of Reovirus and *Candida glabrata* proteins Epa 1, 6, and 7 (Mandelboim et al., 2001; Vitenshtein et al., 2016; Bar-On et al., 2017). NKp46 is a natural cytotoxicity receptor, also called NCR1 and is known once it engages its ligand (NCR1-ligand) to lyse target cells (Narni-Mancinelli et al., 2012). NKp46 can also promote the expansion and survival of NK cells similar to other activating receptors (Lee et al., 2009; Narni-Mancinelli et al., 2012). NKG2A is an inhibitory receptor that recognizes non-classical MHC Class I known as Qa-1b (Vance et al., 1998; Holderried et al., 2013). NKG2A prevents NK cell activation. Based on the licensing paradigm and our data we hypothesized that during chronic *T. gondii* infection, there was an increase in non-self (NCR1-ligand) while there was a decrease in self (Qa-1b) which in turn caused NK cells to negatively regulate CD8+ T cells resulting in parasite reactivation and death. To test this hypothesis spleens from chronically infected mice at weeks 5 and 7 post infection were isolated and the expression levels of NKp46-ligand and Qa-1b were measured on total splenocytes and CD8+ T cells and compared to naïve animals. NCR1-ligand was detected using soluble murine NCR1 (NKp46) fused to human Fc and Qa-1b using anti-Qa-1b antibody. In naïve mice total splenocytes were positive for Qa-1b and largely negative for ligands that were bound by NCR1 (Figure 7A, top and bottom panels, respectively). At week 5 post infection Qa-1b was significantly increased in expression and NCR1-ligand remained low compared to naïve mice. At week 7 post infection Qa-1b expression was decreased significantly compared to week 5 and naïve animals while NCR1-ligand was increased significantly (Figure 7A). As shown in Figure 7B, this pattern of Qa-1b and NCR1-ligand expression was similar when CD8+ T cells were gated and assessed. However, the changes in Qa-1b and NCR1-ligand did not appear to be greater on CD8+ T cells than total splenocytes. We performed preliminary assessments of whether the NK cells were actually more cytotoxic, but did not find any significant increase (data not shown), suggesting these interactions were promoting NK cell survival and maturation as measure by KLRG1 expression on the NK cells (Figure 5A). Overall the decrease in self (Qa-1b) and the increase in non-self (NCR1-ligand) support the concept that NK cell licensing was contributing to CD8+ T cell dysfunction in some way. Therefore, to test that these NK cells via a licensing process were contributing to immune dysfunction during chronic *T. gondii* infection, we infected animals as before and starting at week 5 post infection we treated or not mice with non-depleting anti-NKp46 blocking antibody (Narni-Mancinelli et al., 2012). This approach would not deplete NK cells, but simply block the interaction between NKp46 and the unknown ligand thus potentially decrease NK cell negative regulation of CD8+ T cells. As shown in Figure 7C, anti-NKp46 significantly prolonged the life of mice with chronic *T. gondii* infection compared to no treatment controls. We also wanted to test how blocking NKp46 could impact the frequency of NKG2A+ NK cells in chronically infected mice. As shown in Figure 7D, the frequency of NK1.1+ NKG2A+ cells was significantly decreased when NKp46 was blocked compared to 1 X PBS treated controls. These results suggest that modifications of self vs. non-self and NK cell recognition of these modifications via NKp46 and NKG2A receptors potentiate NK cell dependent negative regulation of CD8+ T cells responses during chronic *T. gondii* infection. The interaction of NK cells with NCR1 ligand may help promote the development of this population of cells. NK cells as a result contribute to immune exhaustion not early during infection, but later after chronic *T. gondii* infection is established.

## Discussion

The immune mechanisms regulating CD8+ T cell exhaustion resulting in reactivation of chronic *T. gondii* infections are poorly understood. In this study we sought to further explore these mechansisms and proposed that NK cells could contribute to this process. NK cells are innate immune cells and belong to a growing family of immune cells known as innate lymphoid cells (ILCs) (Spits et al., 2013; Eberl et al., 2015). NK cells provide a first of defense against many pathogens via their ability to lyse tumor cells and infected cells and produce high levels of IFNγ. Although they have a primary role in innate immune protection, they can also contribute to long-term immunity. NK cells participate in memory responses by further differentiating and developing long life and more efficient recall responses (O'Leary et al., 2006; Cooper et al., 2009; Sun et al., 2009). During acute viral infections, systemic infections, and in the tumor microenvironment NK cells can dysregulate CD4+ and CD8+ T cell responses promoting pathogen and tumor persistence and immune exhaustion (Perona-Wright et al., 2009; Lang et al., 2012; Waggoner et al., 2012, 2014; Cook and Whitmire, 2013; Crouse et al., 2014; Schuster et al., 2014; Cook et al., 2015; Crome et al., 2017). In addition NK cells can become exhausted themselves in different tumors models and infection (Sun et al., 2015). Based on this published knowledge of the complexity of NK cell biology, we tested whether NK cells become exhausted during chronic *T. gondii* infection, how they impact long term immunity to the chronic stage of infection and the mechanisms involved. Our studies demonstrate that NK cells do not appear to become exhausted because their numbers are stable and they do not increase PD1 or LAG3 expression despite losing the ability to produce IFNγ. They appear to alter chronic infection load in brain and erode secondary immune responses in chronically infected animals. They may accomplish this by increasing CD8+ T cell apoptosis in the periphery and as a result decrease the number of protective CD8+ T cells in both spleen and brain. We did not measure CD8+ T cell apoptosis in brain where many antigen specific cells exist. However, since these cells are likely primed in secondary lymphoid organs what we observe in the spleen apoptosis may reflect what is occurring in other tissues. This will be clarified by future studies. NK cells have increased activation as indicated by high KLRG1 and CD107a expression. During chronic *T. gondii* infection NK cells develop a unique Lin-CD49b+CD49a–Ly49–NKp46+CD94+NKG2A+ phenotype in spleen and brain suggesting that these cells receive signals from altered self through NKp46 recognition of specific ligands and a reduction in Qa-1b. Indeed staining of total spleen and CD8+ T cells with soluble NCR1 and anti-Qa-1b indicate there is a significant change in altered self during chronic *T. gondii* infection. Our studies further support this hypothesis when we block NKp46 interaction and rescue chronically infected mice from death caused by CD8+ T cell exhaustion similarly to depletion of NK cells. This blockade also reduces the frequency of NKG2A+ NK cells in spleen. Overall we find that NK cells are essential for acute immune protection by helping to control the parasite with IFNγ and also by helping to prime CD8+ T cells. However, during chronic *T. gondii* infection NK cells develop a response that contributes to CD8+ T cell dysfunction thereby promoting chronic infection in mice.

NK cells can develop immune exhaustion in the tumor microenvironment, after overstimulation and during HCV infection (Gill et al., 2012; Sun et al., 2015; Alvarez et al., 2019; Zhang et al., 2019). Our results suggest that NK cells are not becoming exhausted, but are developing into cells that negatively regulate the CD8+ T cell responses during chronic *T. gondii* infection. CD8+ T cells are known to develop immune exhaustion during chronic *T. gondii* infection (Bhadra et al., 2011b, 2012). This leads to the reactivation of encysted parasites in the CNS as indicated by reduced cyst numbers in brain and increased parasites in the peripheral tissues such as blood and spleen and ultimately results in death of B6 mice (Bhadra et al., 2011b; Hwang et al., 2016). We observe higher cyst numbers in the brains of NK cell depleted animals compared to non- depleted groups during chronic *T. gondii* infection. These data could suggest that NK cells are promoting the reactivation of chronic infection and that cysts are maintained better because there are higher numbers of functional CD8+ T cells in the brain. However, we have yet to thoroughly investigate whether there is an increase in parasite reactivation indicated by reemergence of parasitemia or life stage transformation from bradyzoite to tachyzoite in the brain. The higher cysts numbers we observe may be counterintuitive suggesting a potential different mechanism. The depletion of NK cells could increase the amount of parasite antigen due to lack of control of the parasite resulting in higher cysts numbers and CD8+ T cell responses. We have not ruled this out in this study and future investigation will be carried out to completely understand the mechanisms involved.

Exhausted CD8+ T cells have defective secondary recall responses to parasite challenge (Bhadra et al., 2011b). The maintenance of polyfunctional memory CD8+ T cells during chronic *T. gondii* infection wanes, likely resulting in loss of this long-term protective cell population (Bhadra et al., 2012). Other adaptive immune cells such as B cells may also contribute to long term immunity to the parasite and it is unclear at this time whether they also become exhausted (Kang et al., 2000; Sayles et al., 2000). This raised the question about how the presence of NK cells might contribute to the waning of long-term immunity to the parasite and how this might impact protection against a secondary challenge. Our results indicate that NK cell depleted animals were better able to survive a lethal secondary challenge with either Type II or Type I strain infections. One possible explanation for this result is that NK cells may negatively impact secondary memory CD8+ T cell responses, CD4+ T cell responses or memory B cell responses to the parasite. Another possibility to explain our results is that NK cells and/or ILC1, which are also targeted by our antibody depletion, could contribute to the death observed by increasing immune pathology associated with the secondary challenges (Klose et al., 2014). How NK cells are decreasing secondary responses are still unclear and this question will be addressed in future studies.

CD8+ T cell exhaustion during chronic *T. gondii* infection is marked by reduced CD8+ T cell numbers, decreased frequencies and numbers of IFNγ+CD8+ T cells in the spleen and brain, increased CD8+ T cell apoptosis and high expression of PD1 on the surface of CD8+ T cells. These are hallmarks of CD8+ T cell exhaustion in several infection and disease models (Wherry and Kurachi, 2015). Our results demonstrate that NK cells are present in the spleen and brain during chronic *T. gondii* infection, they do not have reduced numbers and do not express high levels of PD1 or LAG3 as compared to CD4+ and CD8+ T cells. When we investigated NK cell function (IFNγ and CD107a), we observed that although NK cells in chronic *T. gondii* infection lose the ability to produce IFNγ, they increase their CD107a expression indicating a gain of function. Moreover, NK cell depletion rescued mice from death during chronic infection. NK cell depletion helped restore CD8+ T cell function in spleen and brain of chronically infected animals and enhanced the survival of chronically infected mice after secondary parasite challenge. Our results suggest that one way NK cells may affect CD8+ T cell immunity to the parasite is by increasing their apoptosis. We only measured apoptosis in the spleen, whether NK cells also increase CD8+ T cell apoptosis in the brain is unclear. Although, NK cells may lose the ability to produce IFNγ, our results suggest that unlike tumor, overstimulation and persistent HCV infection (Gill et al., 2012; Sun et al., 2015; Alvarez et al., 2019; Zhang et al., 2019), they are also gaining function that negatively regulates the adaptive response to chronic *T. gondii* infection. The mechanism by which they are causing this negative regulation is unclear and will be important in future studies.

NK cells in the steady state express a stochastic array of activating and inhibitory receptors that help regulate their function (Pegram et al., 2011; Kruse et al., 2014; Sun, 2016). In mice this includes the Ly49 family of receptors (D-I), natural cytotoxicity receptors (NCRs), NKG2D, 2B4, and CD94/NKG2A. In the naïve state in B6 mice, these receptors are expressed on most NK cells in different combinations, but at relatively high frequencies. Our data demonstrates that NK cells during chronic *T. gondii* infection have altered expression of NK cell receptors. We observe a near complete loss of Ly49 D, H and I. At the same time we observed the maintenance of CD49b+ NKp46+ NK1.1+ cells. Within this population the frequency of CD94+NKG2A+ cells increased dramatically and this increase was only observed during persistent chronic *T. gondii* infection and not after infection with the non-persistent vaccine strain. There are many reasons why these NK cells develop such a different phenotype from conventional NK cells during chronic *T. gondii* infection. One reason could be due to the chronic inflammatory environment of the mice. In the absence of common gamma chain cytokines, NK cells can be developed via an IL-12 bypass pathway (Ohs et al., 2016). The NK cells that develop via this emergency NK cell lymphopoiesis pathways exhibit low level expression of Ly49 proteins and are enriched for CD94+NKG2A+ cells. A recent study has demonstrated that during *T. gondii* Prugniaud and attenuated *cps1-1* strain infection, NK cells are plastic and could differentiate into modified NK/ILC1 type cell that persist with memory-like features (Park et al., 2019). As we have previously published, in our hands NK cells do not persist during *T. gondii* infection (Ivanova et al., 2019). However, the NK/ILC1 like cells observed in the previous study exhibit a similar receptor phenotype to those we observe in this study during chronic ME49 infection. The development of the NK/ILC1 like cell may be indirectly STAT4 dependent, but not through cell intrinsic IL-12 induced signaling. This could indicate the role of different continually expressed cytokines such as IFNγ, IL-6, or TGFβ during chronic *T. gondii* infection. The role of transcription factors Tbet and Eomes were also not clear in the previous study (Park et al., 2019). Whether the cells we observe and are calling NK cells are truly NK cells is therefor still an outstanding and complex question to be addressed. Given the level of ILC plasticity it is entirely possible that the cells we are studying are ILC1 or ILC3. We have not fully investigated transcription factor expression in these cells but are planning this for future studies. Therefore we hypothesize that the cells we observe are NK cells being generated de novo after chronic infection is established as a result of the chronic inflammatory environment. Our future studies are examining this possibility.

NK cells appear to have a licensed NK cell phenotype during chronic *T. gondii* infection because of the presence of both activating and inhibitory receptors on their surface (Kim et al., 2005). Thus, the licensing paradigm could also explain why this phenotype of NK cells develops during chronic *T. gondii* infection. In this situation, the chronic infection environment in Toxoplasmosis causes the activating receptor NKp46 to recognize a ligand expressed on target cells that potentiates the activation, survival and increased abundance of NKp46+ CD94+NKG2A+ NK cells. This is what occurs with other activating receptors including Ly49H after it recognizes m157 from MCMV (Lanier, 2005; Sun et al., 2009). As a result of Ly49H and m157 interaction, Ly49H+ NK cells are more abundant, have longer life and can respond more efficiently to secondary infection. While Ly49H interaction with MCMV m157 could directly activate all Ly49H positive NK cells regardless of inhibitory receptor expression, our data suggests that because of the higher frequency of CD94+NKG2A+ NK cells within the NKp46+ population, that loss of the inhibitory signal through NKG2A could also help promote the development of this NK cell population. What NKp46 could be recognizing is still a mystery during chronic *T. gondii* infection. The ligands for NKp46 vary in source and structure and to date may include Influenza virus HA, Sigma 1 protein of Reovirus and *Candida glabrata* proteins Epa 1, 6, and 7 (Mandelboim et al., 2001; Vitenshtein et al., 2016; Bar-On et al., 2017). Our data indicate that there is increased staining of spleen cells and CD8+ T cells with soluble NCR1. We also observed reduced Qa-1b expression. Moreover, blockade of NKp46 with a non-depleting anti-NKp46 antibody rescued mice to a similar level from death compared to NK cell depletion with anti-NK1.1. What protein modifications are occurring or genes that are being expressed to produce NCR1 ligand are unclear, however, the increase in binding of soluble NKp46 and anti-NKp46 blockade supports the hypothesis that the NKp46 signal is required for the development of this unique NK cell population during chronic *T. gondii* infection.

NKG2A is an inhibitory receptor expressed on NK cells and CD8+ T cells that recognizes the non-classical MHC protein Qa-1b, which presents the leader sequence of classical MHC Class I (Vance et al., 1998). Through NKG2A-Qa-1b interactions NK cell lysis of target cells and IFNγ production can be inhibited (Colmenero et al., 2007; Lu et al., 2007). Qa-1b expression by B cells during high dose LCMV infection can limit NK cells negative regulation of LCMV specific T cell responses (Xu et al., 2017). Blocking this interaction results in higher viral persistence. NKG2A interaction with Qa-1b can also limit the activation levels of virus specific CD8+ T cells in acute poxvirus infection resulting in reduced activation induced cell death and better antigen specific CD8+ T cell responses and control of virus (Fang et al., 2011; Rapaport et al., 2015). Interestingly, Qa-1b also is important for the development of protective non-conventional CD8+ T cell responses in the salivary gland of mice during acute MCMV infection in the absence of conventional CD8+ T cells (Anderson et al., 2019). Much of what is understood about NKG2A-Qa-1b interactions is in the context of acute infections. We present data that at WK5 post infection with *T. gondii*, Qa-1b is increased in expression. However, by WK7 post infection Qa-1b expression decreases. Along with increased NCR1-ligand expression at WK 7 post infection, the loss of Qa-1b expression could promote the negative regulation of CD8+ T cell responses by NK cells to promote chronic *T. gondii* infection. Therefore our data supports the hypothesis that during *T. gondii* infection the role of NKG2A-Qa-1b axis is to keep NK cells from eroding CD8+ T cell responses important for control of the parasite. Whether the loss of this interaction is important for the development of these NK cells during chronic *T. gondii* infection is still unclear. We also have not examined how NKG2A-Qa-1b interactions impact immunity during acute *T. gondii* infection. Other receptors could also impact the NK cell responses we observe during chronic *T. gondii* infection. These include DNAM1 since DNAM1 expression on NK cells can limit immature DC (Seth et al., 2009). NK cells with high DNAM1 expression also had low level expression of Ly49 markers in mice. The role of DNAM1 could be further explored in future studies.

Another important phenotype we observe is the increase in KLRG1+ NK cells in chronically infected mice. KLRG1 is an inhibitory receptor expressed more highly as NK cells mature (Tessmer et al., 2007; Geiger and Sun, 2016). NK cell maturation is activating receptor dependent. Recent studies investigating exhausted NK cells during chronic stimulation suggest that increased KLRG1 indicates NK cell exhaustion (Alvarez et al., 2019). In this study, NKG2D interaction with high levels of NKG2D ligands results in increased KLRG1 expression and loss of NKG2D expression on the cells and NK cell exhaustion. Therefore, another possible explanation of the development the phenotype of NK cells during chronic *T. gondii* infection could be that ligands for other receptors are highly upregulated during chronic *T. gondii* infection. This could then explain why we observe a loss of expression of Ly49 D, Ly49H, and Ly49I+ NK cells. However, we performed an exhaustive analysis of known murine NK cell receptor ligands and we did not detect any increase in their expression during chronic *T. gondii* infection (data not shown).

Our data demonstrates that NK cells present during chronic *T. gondii* infection alter their role in immunity and act as negative regulators of CD8+ T cells to promote reactivation of the parasite. NK cells are the cytotoxic ILC (Cortez and Colonna, 2016). They also produce high levels of IFNγ and other cytokines after activation. NK cells are usually considered to be a first line of defense against many pathogens and tumors. However, many recent reports demonstrate that NK cells can also negatively regulate adaptive immune responses through several different mechanisms (Perona-Wright et al., 2009; Lang et al., 2012; Waggoner et al., 2012, 2014; Cook and Whitmire, 2013; Crouse et al., 2014, 2015; Schuster et al., 2014; Cook et al., 2015; Rydyznski et al., 2015; Crome et al., 2017; Kwong et al., 2017). These include the production of the immunosuppressive cytokine IL-10. NK cells are activated to produce IL-10 during acute stage systemic infections including *T. gondii*. NK cells can also induce apoptosis or kill CD4+ and/or CD8+ T cells during acute infections through TRAIL-TRAILR interactions, NKp46 dependent cytotoxicity and cytotoxicity through undefined receptor ligand pairs. NK cells can also kill tumor infiltrating lymphocytes (TILs) via an NKp46 dependent process. Another study recently published suggests that NK cells that become exhausted during persistent HCV infection lose their ability to produce IFNγ and as a result the CD8+ T cell effector population is unable to be maintained (Zhang et al., 2019). These studies suggest that NK cells can secrete immune suppressive cytokines to act systemically to suppress immunity, can act directly against T cells and kill them or because they are exhausted themselves they are unable to help maintain CD8+ T cell functions. During chronic *T. gondii* infection we observe that NK cells lose their ability to produce IFNγ while increasing the CD107a expression. Thus, while NK cells might lose one function during chronic *T. gondii* infection they appear to have a gain of function. We did attempt to measure whether NK cells from chronically infected mice were more cytotoxic, but we did not observe any increase (data not shown). Importantly CD107a is only a surrogate marker for NK cell cytotoxicity (Alter et al., 2004). CD107a can associate with other secretory vesicles and in particular can be surface expressed alongside MHC Class II on DCs (Alter et al., 2004; Michelet et al., 2015). Therefore, we believe that the increase in CD107a on NK cells during chronic *T. gondii* infection may indicate a different type of immune suppressive function. What that suppressive function might be is still unclear. We did not observe NK cells producing IL-10 during the chronic stage of parasite infection and they also did not increase their expression of PDL1, the ligand for PD1. PDL1 expression can promote exhaustion of CD8+ T cells during chronic *T. gondii* infection (Bhadra et al., 2011b). Therefore, based on our results we propose that NK cells are acquiring a different type of immune suppression than producing IL-10 or being cytotoxic. Another possibility is that sustained NK cell IFNγ is required to help maintain CD8+ T cell function during chronic *T. gondii* infection. NK cells are thought during acute *T. gondii* to help prime CD8+ T cell responses, especially in the absence of CD4+ T cell help (Combe et al., 2005). We confirmed the importance of NK cells for priming CD8+ T cells in this study. Based on these studies and our data along with data from the persistent HCV infection study, a lack of NK cell IFNγ could also not support CD8+ T cell function. However, our data show that NK cell depletion enhances CD8+ T cell function during chronic *T. gondii* infection making this less likely and that the negative regulation of CD8+ T cell responses by NK cells is via a different mechanism.

The results from our study open a question about where NK cells might negatively regulate CD8+ T cell responses during chronic *T. gondii* infection. We observe NK cells present in both spleen and in brain. Brain numbers are very low and we did not measure whether NK cells and CD8+ T cells came into contact in the spleen or brain during chronic *T. gondii* infection. Since ILCs can traffic to the CNS in EAE (Kwong et al., 2017) and cause pathology, one potential tissue where NK cells might decrease CD8+ T cell function is in the CNS. This has yet to be tested. Another possible location could be in secondary lymphoid organs where CD8+ T cells are receiving signals to traffic to the CNS. This will require extensive future studies to understand where NK cells are impacting CD8+ T cells during chronic *T. gondii* infection.

In this study we present data suggesting that during chronic *T. gondii* infection, NK cells are still present, do not appear exhausted based on cell number and PD1 or LAG3 expression. They negatively impact the mortality of chronically infected mice and NK cell depletion rescues animals during CD8+ T cell exhaustion (CD8+ T cell function is maintained and apoptosis reduced in the spleen). The NK cells develop a unique phenotype and are enriched for cells that are CD49b+ NKp46+ CD94+ NKG2A+ KLRG1+. The development of this population could be dependent upon activating receptor NKp46 recognition of a specific ligand while NKG2A interaction with Qa-1b is reduced. NK cells suppress the CD8+ T cell response by an as of yet identified mechanism that may be independent of cytotoxicity, IL-10 production or the expression of PDL1. Overall in chronic *T. gondii* infection, NK cells may contribute to CD8+ T cell exhaustion and persistence of the parasite and manipulating them to prevent the development of this response could improve health outcomes for individuals susceptible to parasite reactivation.

## Data Availability Statement

The datasets generated for this study are available on request to the corresponding author.

## Ethics Statement

The animal study was reviewed and approved by the University of Wyoming Institutional Animal Care and Use Committee (IACUC) (PHS/NIH/OLAW assurance number: A3216-01) approved all animal protocols.

## Author Contributions

RK and JG developed the scientific concept. RK, DI, SD, KF, GS, DR, RF, TM, JM, and JG carried out experiments, acquired and analyzed data, and helped generate figures for the manuscript. JG wrote the manuscript. ID consulted on the manuscript. All authors contributed to the article and approved the submitted version.

## Conflict of Interest

The authors declare that the research was conducted in the absence of any commercial or financial relationships that could be construed as a potential conflict of interest.
